# Synthesis of Pregnane Derivatives, Their Cytotoxicity on LNCap and PC-3 Cells, and Screening on 5α-Reductase Inhibitory Activity

**DOI:** 10.3390/molecules14114655

**Published:** 2009-11-17

**Authors:** Sujeong Kim, Eunsook Ma

**Affiliations:** College of Pharmacy, Catholic University of Daegu, Hayang, 712-702, Korea

**Keywords:** 5α-reductase inhibitor, LNCaP, PC-3, epoxypregnanes, 20-oxime pregnanes

## Abstract

A series of epoxy- and/or 20-oxime pregnanes were synthesized from commercially available pregnenolone. Compounds **1**, **3**, **7**, **8** and **11-13** were evaluated for cytotoxicity activity towards LNCaP (androgen-dependent) and PC-3 (androgen-independent) prostate cancer cells. Compound **13** showed the highest activity on both LNCaP (IC_50_ 15.17 μM) and PC-3 (IC_50_ 11.83 μM) cell lines. Compound **11** showed weak activity on LNCaP cells (IC _50_ 71.85 μM) and **8** showed the weak activity on PC-3 cells (IC_50_ 68.95 μM), respectively. The 5α-reductase II (5AR2) inhibitory effects of compounds **1**-**3**, **5** and **7**-**13** were investigated in a convenient screening model, in which compounds **5**, **8**, **11** and **12** were observed to be potential inhibitors of 5α-reductase, in particular, the 4**-**azasteroid **11,** that also inhibited cell proliferation of androgen-dependent cells and **8,** that in addition inhibited PC-3 cells more potently than LNCaP cells.

## 1. Introduction

Testosterone (T) and dihydrotestosterone (DHT) play a key role in the maintenance of cell proliferation in the prostate gland. The enzyme steroid 5α-reductase (5AR) catalyzes the NADPH-dependent reductive conversion of T to DHT. The deficiency of 5AR in males results in an incomplete differentiation of external genitalia at birth [1]. On the other hand, abnormally high 5AR activity in humans results in excessively high DHT levels in peripheral tissues, which is implicated in the pathogenesis of prostate cancer, benign prostatic hyperplasia (BPH), acne and male pattern baldness [2]. It is presently recognized that there are two genes encoding two distinct isozymes of 5AR that are differentially expressed in human tissues and referred to as type 1 5AR (5AR1) and type 2 5AR (5AR2) [3,4,5]. Although 5AR1 is predominantly expressed in the skin and liver, 5AR2 is mainly expressed in prostate, seminal vesicles, liver and epididymis [6].

Various steroidal [7,8,9] and non-steroidal [10,11] inhibitors have been synthesized and tested against 5AR. Of these, finasteride (PROSCAR^®^), a type II-selective 5α-reductase inhibitor, was the first 5AR inhibitor approved in the USA for the treatment of BPH and prostate cancer. Finasteride was also reported to reduce the proliferation rate *in vitro* of DU145 and PC-3 prostate cancer cell lines, although several reports in the literature classified them as being “hormone-independent” [12]. Bologna reported that the growth rate of the LNCap cell line can be dose-dependently inhibited by a 5α-reductase inhibitor (finasteride) and antiandrogens (cuproterone and hydroxyflutamide) under culture conditions with 5% FCS DMEM; on the other hand, growth of these cells is only modestly stimulate by T and by DHT when those hormones are added under the same culture conditions [13]. However, finasteride is slow acting and produces side effects affecting sexual function [14,15]. It has also been demonstrated that dutasteride acts as a 5AR1 and 5AR2 inhibitor and blocks the activation of the androgen receptor with the consequences of decreased proliferation and increased death of LNCaP cells [16], but is unresponsive on androgen-independent PC-3 cells [17].

Some steroidal 20-oximes were reported as dual inhibitors of P450 17 (17α-hydroxylase/C17,20-lyase) and 5α-reductase [7,18]. 3-Acetoxy-5α,6α-epoxyprognan-16-ene-20-one, which doesn’t have a double bond in the A or B rings [19], and 6α,7α-epoxy progesterone [8], showed a higher 5AR inhibitory activity than finasteride. Some steroidal epoxy compounds have been used as important intermediates to synthesize potent inhibitors of 5α-reductase, since their facile ring opening allows the introduction of various functionalities in a stereospecific manner [20].

On the basis of the facts described in these references and in order to obtain the skeleton structure required for 5AR inhibitory activity, we describe here the synthesis of some 20-oximes and/or epoxy pregnane, pregnene and pregnadiene derivatives, their *in-vitro* cytotoxicity on LNCaP (androgen-dependent) and PC-3 (androgen-independent) prostate cancer cells, and the screening test results as potential inhibitors of 5AR2.

## 2. Results and Discussion 

### 2.1. Synthesis of steroidal compounds

**Scheme 1:**
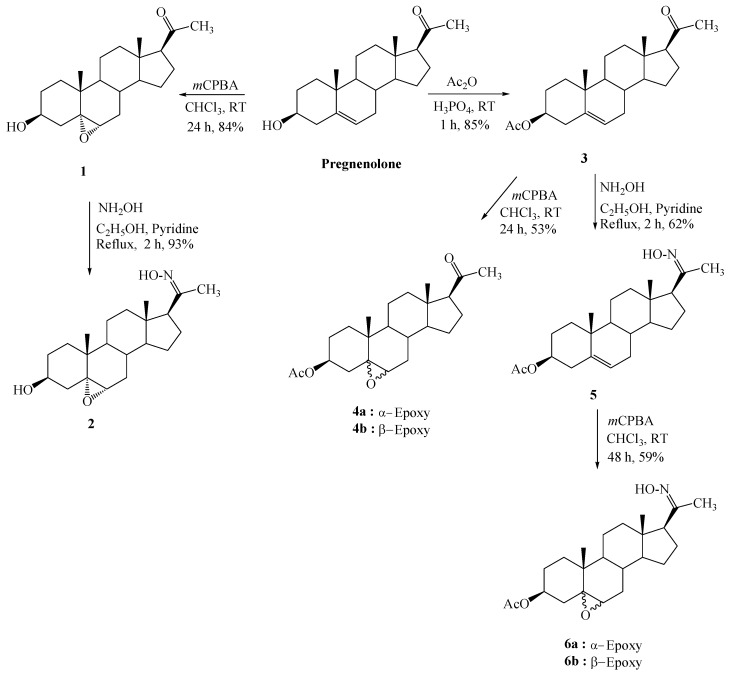
Synthesis of 5,6-epoxy- and/or 20-oxime derivatives.

The synthesis of the eight 5,6-epoxy- and/or 20-oxime pregnane or pregnene derivatives is shown in Scheme 1. Pregnenolone was reacted with *m*-chloroperoxybenzoic acid (*m*CPBA) to form 5α,6α-epoxy-3β-hydroxypregnan-20-one (**1**). The α-epoxide stereochemistry of compound **1** was confirmed by irradiation of H-6 (2.92 ppm), which showed a NOE to the C-19 methyl protons (1.06 ppm) in the 1D-NOESY spectrum. Compound **1** was quantitatively converted to the 20-oxime analog **2** using hydroxylamine.

Pregnenolone was also further reacted with acetic anhydride to give 3β-acetoxy-5-pregnen-20-one (**3**), which was reacted with *m*CPBA to afford a 1:1 mixture of 5α,6α- and 5β,6β-epoxy-3β-acetoxypregnan-20-one (**4a** and **4b**). The α- or β-configurations of the 5,6-epoxy group of **4a** and **4b** were identified by irradiation of H-6 (3.10 and 2.91 ppm) which resulted in a NOE to the C-19 methyl protons (1.08 ppm) in the 1D-NOESY spectrum. The 1D-NOESY spectrum showed that only H-6 (3.10 ppm) was correlated with the C-19 methyl protons. Consequently, this compound was determined as 5α,6α-epoxy-3β-acetoxypregnan-20-one (**4a**) and the other H-6 proton (2.91 ppm) did not show the correlation, it was identified as 5β,6β-epoxy compound **4b**. Compound **3** was reacted with hydroxylamine to give 3β-acetoxy-20-hydroxyimino-5-pregnen-20-one (**5**), which was reacted with *m*CPBA to produce a mixture of 5α,6α- and 5β,6β-epoxy-3β-acetoxy-20-hydroxyiminopregnan-20-ones (**6a** and **6b**).

**Scheme 2:**
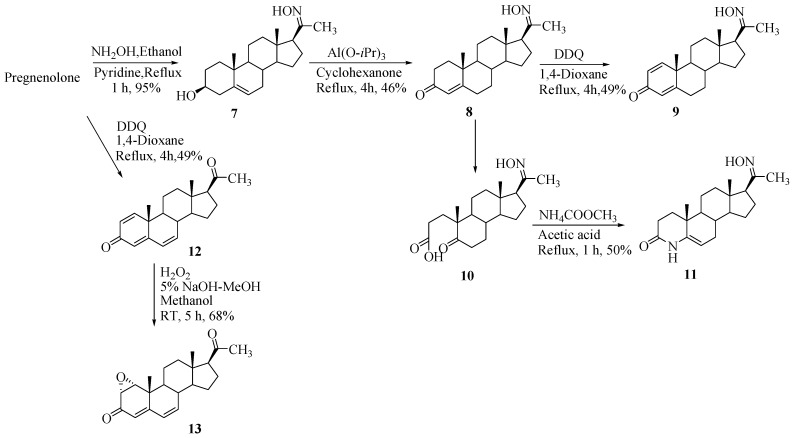
Synthesis of 4-azasteroid **11** and unsaturated steroid derivatives.

The synthesis of the 4-azasteroid **11** is shown in Scheme 2. Pregnenolone was reacted with hydroxylamine to give 3β-hydroxy-20-hydroxyiminopregnenolone (**7**), which was oxidized with aluminum isopropoxide in cyclohexanone to yield 20-hydroxyimino-4-pregnene-3,20-dione (**8**) by Oppenauer oxidation. Compound **8** was oxidized with 2,3-dichloro-5,6-dicyano-1,4-benzoquinone (DDQ) to form 20-hydroxyimino-1,4-pregnadiene-3,20-dione (**9**) by a reported method [21]. Ring cleavage of **8** by KMnO_4_ and NaIO_4_ gave 20-hydroxyimino-5-oxo-A-nor-3,5-secopregnan-3-oic acid (**10**), which was cyclized with ammonium acetate to yield 4-aza-20-hydroxyimino-5-pregnene-3,20-dione (**11**). The structure assignment of **10** was made by the disappearance of the double bond proton (H-4) in the corresponding ^1^H-NMR spectrum and by the appearance of two carbonyl carbons (214.7 and 209.2 ppm) in the ^13^C-NMR spectrum. 4-Azasteroid **11** was identified by observing a double bond proton (H-6, 4.82 ppm) and an NH proton (7.41 ppm) in the ^1^H-NMR spectrum and one carbonyl carbon (209.3 ppm) in the ^13^C-NMR spectrum. 1,4,6-Pregnatriene-3,17-dione (**12**) was synthesized from pregnenolone with DDQ and then reacted with 30% H_2_O_2_ in 5% NaOH-MeOH to give 1α,2α-epoxy-4,6-pregnadiene-3,17-dione (**13**), stereoselectively. The α-epoxy configuration of the 1,2-epoxy group in **13** was determined from the 1D-NOESY spectrum, where irradiation of the H-1 proton (4.16 ppm) showed an NOE to 19-CH_3_ (1.19 ppm). 

### 2.2. MTT test

Human prostate carcinoma cell lines have been available since 1977 and represent a good experimental model to assess new hormonal therapies [12]. Charcoal stripped-dextran treated heat inactivated 10% FBS was used for these assays to remove the endogeneous steroids.

**Figure 1:**
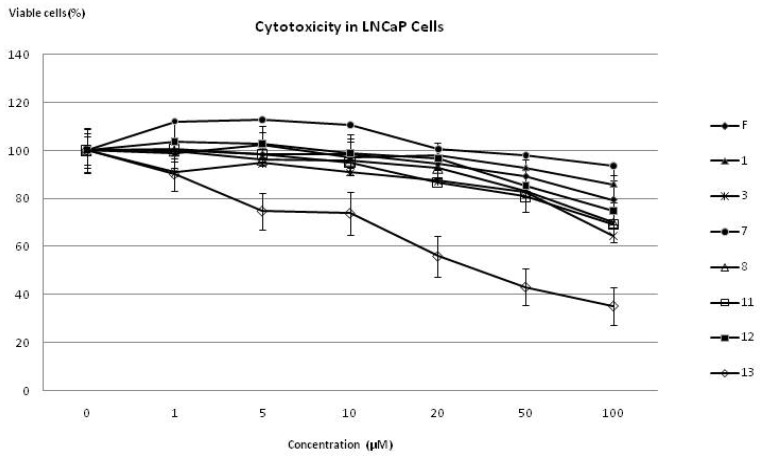
Cytotoxicity of F (finasteride) and **1**, **3**, **7**, **8**, **11**-**13** on LNCaP cells.

**Figure 2:**
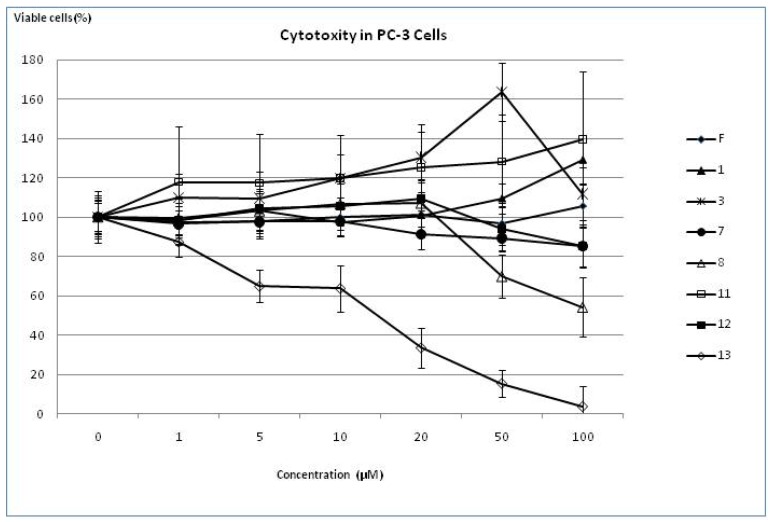
Cytotoxicity of F (finasteride) and **1**, **3**, **7**, **8**, **11**-**13** on PC-3 cells.

We assessed the cytotoxicity of synthesized compounds **1**, **3**, **7**, **8** and **11-13** on LNCap (androgen-dependent) and PC-3 (androgen-independent) cancer cells. (Figure 1 and Figure 2) As shown in Figure 1, the 4-azasteroid **11** showed a dose dependently inhibitory effect on LNCaP cell and also exerted a higher cytotoxicity effect than finasteride, but **8** inversely inhibited the cell proliferation of PC-3 cell. 1,2-Epoxy compound **13** showed the highest cytotoxicity on both LNCaP (IC_50_ 15.17 μM) and PC-3 (IC_50_ 11.83 μM) cells. (Table 1) We thought that the cytotoxicity ability of **13** was due to epoxy ring especially on 1,2-position of steroid structure independent of cell type.

### 2.3. Screening test of 5α-reductase II inhibitory activity

**Figure 3:**
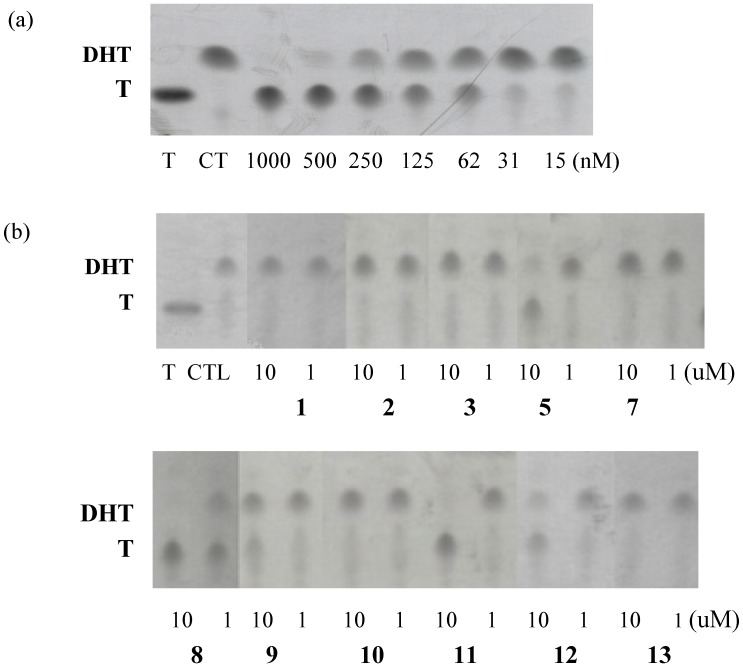
Data of type II 5α-reductase inhibitory activity of finasteride (a) and synthetic compounds (b). Ten micrograms of stable cell extracts was incubated with 1 μCi [^3^H]testosterone and synthetic compounds (1 μM and 10 μM) for 1 hr. T, testosterone; DHT, 5α-dihydrotestosterone.

This purpose of this experiment is to obtain preliminary data on the 5AR2 inhibitory activity of the synthetic compounds. Jang *et al*. [22] reported that the HEK293 cells were stably transfected with 5AR2 and they suggested RT-PCR data to validate that the stable transfection has been achieved. They determined that 10 μg of stable cell extract completely converted 1 μCi of T into DHT in an *in vitro* enzymatic assay. We used this convenient screening method to observe the 5α-reductase inhibitory activities of compounds **1**, **2**, **3**, **5** and **7**-**13**. As shown in Figure 3, 3β-acetoxy-20-hydroxyimino-5-pregnen-20-one (**5**), 20-hydroxyimino-5-pregnen-20-one (**8**), 4-aza-20-hydroxyimino-5-pregnene-3,20-dione (**11**) and 1,4,6-pregnatriene-3,20-dione (**12**) showed potential 5α-reductase inhibitory activity. None of the 3-hydroxy pregnene and pregnadiene derivatives showed any inhibitory activity.

The inhibitory mechanism of enone compounds **8** and **12** towards the enzyme 5α-reductase can be generally explained by the fact that 5AR forms an enzyme-steroid activated complex in the first step and the nucleophilic portion of the enzyme attacks their double bond instead of testosterone in a Michael type addition reaction and NADPH subsequently donates a hydrogen to the C-5 position of the compounds [1]. It is assumed that the carbonyl group of the 3-acetoxy substituent of **5** and the 3-carbonyl group of **11** bind to the enzyme and then next step is same as that of the enones. However all tested compounds showed lower activity than finasteride, therefore a substituent at the C-20 position more bulky than an oxime group seems to be required. Consequently, the carbonyl group at the C-3 position, a substituted oxime at the C-20 position and an unsaturated bond in the A and/or B rings of the pregnane skeleton seem to be important for 5α-reductase inhibitory activity. Compound **8** is more active than the 4-azasteride analog **11**, consequently we don’t think that 4-azasteride is a necessary feature for activity. Compound **11** also showed cytotoxicity on androgen-dependent LNCaP cells, therefore the cytotoxicity of this compound was seems to relate with the androgens.

## 3. Experimental 

### 3.1. General

All non-aqueous reactions were performed under a dry atmosphere of nitrogen. The commercial reagents were purchased from Aldrich, Fluka, or Sigma. Solvents were purified and dried prior to use. Melting points was measured on Thomas-Hoover melting point apparatus and not corrected. ^1^H-, ^13^C- NMR and NOESY spectra were taken on a Varian 400 MHz spectrometer in DMSO-*d_6_*, D_2_O or CDCl_3_. Chemical shifts (δ) are in parts per million (ppm) relative to tetramethylsilane, and coupling constants (*J*) are in Hertz. IR spectra were determined on a Jasco FT-IR 300E spectrometer as KBr pellets. GC/MS spectra were obtained on Shimadzu QP 5050 and JEOL GC Mate 2 mass spectrometer. MPLC was performed on Yamazen YFLC-AI and an analytical TLC was performed on pre-coated silica gel 60 F_254_ plates (Merck). Solvent systems for TLC were ethyl acetate/*n*-hexane mixtures and 10% MeOH in methylene chloride. Column chromatography was carried out on Merck silica gel 9385 (230-400 mesh) and eluted with ethyl acetate/*n*-hexane mixtures.

*5α,6α-Epoxy-3β-hydroxypregnan-20-one* (**1**): 5-Pregnen-3*β*-ol-20-one (500 mg, 1.58 mmol) and *m*CPBA (818.0 mg, 4.74 mmol) were dissolved in chloroform (50 mL) and stirred at room temperature for 24 hours. The reaction mixture was diluted with H_2_O and washed with aqueous FeSO_4_, saturated NaHCO_3_, H_2_O and the aqueous layer extracted with dichloromethane. The organic layer was dried with anhydrous MgSO_4_, filtered and evaporated to give the crude compound, which was purified by column chromatography (ethyl acetate/*n*-hexane = 1:1) to give 440 mg (84%) of the pure white product **1**. m.p : 176-179 °C, ^1^H-NMR (CDCl_3_) δ: 3.87-3.96 (1H, m, 3-H), 2.92 (1H, d, *J* = 4.4 Hz, 6-H), 2.50 (1H, t, *J* = 8.8 Hz, 17-H), 2.11 (3H, s, 21-CH_3_), 1.06 (3H, s, 19-CH_3_), 0.56 (3H, s, 18-CH_3_); ^13^C-NMR (CDCl_3_) δ: 209.5 (20-C=O), 68.6 (5-C), 65.7 (3-C), 63.4 (17-C), 59.1 (6-C), 57.0, 43.9, 42.5, 39.8, 38.5, 34.9, 32.4, 31.6, 31.0, 29.9, 28.7, 24.2, 22.7, 20.7, 15.9, 13.2; GC-mass (EI) *m/z* : 332 (M)^+^, 314 (M+1-H_2_O)^+^; IR (cm^-1^): 3441 (3-O-H), 1702 (20-C=O).

*5α,6α-Epoxy-3β-hydroxy-20-hydroxyiminopregnan-20-one* (**2**): Compound **1** (300 mg, 0.90 mmol) was dissolved in ethanol (5 mL) and hydroxylamine hydrochloride (100.8 mg, 1.44 mmol) and pyridine (0.15 mL) were added and the mixture was refluxed at 130 °C for 2 hours. The reaction mixture was diluted with H_2_O and extracted with dichloromethane. The organic layer was dried with anhydrous MgSO_4_, filtered, and evaporated to give crude product, which was recrystallized with ethyl acetate and *n*-hexane to give 290 mg (93%) of the pure white product **2**, m.p : 144-147 °C, ^1^H-NMR (DMSO-*d*_6_) δ: 10.35 (1H, s,=NOH), 4.55 (1H, br s, 3-OH), 3.91 (1H, d, *J* = 2.4 Hz, 6-H), 3.77-3.81 (1H, m, 3-H), 1.72 (3H, s, 21-CH_3_), 1.13 (3H, s, 19-CH_3_), 0.55 (3H, s, 18-CH_3_); ^13^C-NMR (DMSO-*d*_6_) δ:155.8 (20-C=NOH), 75.2 (5-C), 66.2 (3-C), 65.3 (6-C), 56.8 (17-C), 55.0, 45.1, 44.0, 41.9, 39.2, 39.0, 35.8, 33.2, 31.5, 30.8, 24.4, 23.3, 21.3, 18.5, 15.9, 13.9; GC-mass (EI) *m/z* : 348 (M+1)^+^, 330 (M+1-H_2_O)^+^; IR (cm^-1^) : 3401 (O-H), 1640 (C=N).

*3β-Acetoxy-5-pregnen-20-one* (**3**): 5-Pregnen-3*β*-ol-20-one (pregnenolone, 1 g, 3.16 mmol) was dissolved in acetic anhydride (20 mL) and phosphoric acid. The reaction mixture was stirred for 1 hour at room temperature and evaporated to remove acetic anhydride to give crude oily product, which was diluted with H_2_O. The reaction mixture was neutralized with saturated NaHCO_3_ (aq.) and extracted with dichloromethane. The organic layer was dried with anhydrous MgSO_4_, filtered, and evaporated to give crude product, which was purified by column chromatography (ethyl acetate/*n*-hexane = 1:1) to give 960 mg (85%) of pure white product **3**, m.p : 147-149 °C,^1^H-NMR (CDCl_3_) δ: 5.38 (1H, d, *J* = 4.8 Hz, 6-H), 4.56-4.65 (1H, m, 3-H), 2.54 (1H, t, *J* = 9.0 Hz, 17-H), 2.13 (3H, s, 21-CH_3_), 2.04 (3H, s, -COCH_3_), 1.02 (3H, s, 19-CH_3_), 0.63 (3H, s, 18-CH_3_); ^13^C-NMR (CDCl_3_) δ : 209.6 (20-C=O), 170.6 (3-OCOCH_3_), 139.7 (5-C), 122.3 (6-C), 73.8 (3-C), 63.7 (17-C), 56.8, 49.9, 44.0, 38.8, 38.1, 37.0, 36.6, 31.8, 31.7, 31.6, 27.7, 24.5, 22.8, 21.4, 21.0, 19.3, 13.2, GC-mass (EI) *m/z*: 359 (M+1)^+^, 298 (M-CH_3_COOH ^+^, IR (cm^-1^) : 1728 (20-C=O), 1704 (3-C=O). 

*3β-Acetoxy-5α,6α-epoxypregnan-20-one* (**4a**) *and 3β-acetoxy-5β,6β-epoxypregnan-20-one* (**4b**): 3*β*-Acetoxy-5-pregnen-20-one (**3**, 100 mg, 0.28 mmol) and *m*CPBA (145 mg, 0.84 mmol) were dissolved in chloroform (10 mL) and the reaction mixture was stirred at room temperature for 24 hours. The reaction mixture was treated as the same method of compound **1**. The crude compound was purified by column chromatography (ethyl acetate/*n*-hexane = 1:5), giving 56 mg (53%) of a mixture of **4a** and **4b**. Compound **4a**: ^1^H-NMR (CDCl_3_) δ: 4.90-5.00 (1H, m, 3-H), 3.10 (1H, d, *J* = 1.8 Hz, 6-H), 2.09 (3H, s, 21-CH_3_), 2.01 (3H, s, 3-COCH_3_), 1.01 (3H, s, H-19), 0.57 (3H, s, 18-CH_3_), ^13^C-NMR (CDCl_3_) δ: 208.4 (20-C=O), 170.9 (3-OCOCH_3_), 71.3 (5-C), 63.4 (3-C), 56.6 (6-C), 56.2 (17-C), 50.9, 42.4, 38.0, 36.7, 36.1, 32.4, 32.1, 29.7, 27.2, 24.4, 22.8, 22.7, 21.9, 21.3, 20.6, 13.2, 13.1; Compound **4b**: ^1^H-NMR (CDCl_3_) δ: 4.72-4.82 (1H, m, 3-H), 2.91 (1H, d, *J* = 3.6 Hz, 6-H), 2.10 (3H, s, 3-COCH_3_), 2.03 (3H, s, 21-CH_3_), 1.08 (3H, s, H-19), 0.60 (3H, s, 18-CH_3_); ^13^C-NMR (CDCl_3_) δ: 208.4 (20-C=O), 170.8 (3-OCOCH_3_), 71.4 (5-C), 63.3 (3-C), 56.7 (6-C), 56.2 (17-C), 50.9, 42.4, 38.0, 36.7, 36.1, 32.4, 32.1, 29.7, 27.2, 24.4, 22.8, 22.7, 21.9, 21.3, 20.6, 13.2, 13.1, GC-mass (EI) *m/z* : 374 (M)^+^, IR (cm^-1^) : 3441 (3-O-H), 1702 (20-C=O).

*3β-Acetoxy-20-hydroxyimino-5-pregnen-20-one* (**5**): 3*β*-Acetoxy-5-pregnen-20-one (**3**, 100 mg, 0.28 mg) was dissolved in ethanol (2 mL), hydroxylamine hydrochloride (31.3 mg, 0.45 mmol) and pyridine (0.1 mL) and the reaction mixture was refluxed for 2 hours at 130 ℃. The reaction mixture was poured into cold water (30 mL) to form a precipitate, which was recrystallized with ethyl acetate and *n*-hexane to afford 65 mg (62%) as the pure white product **5**, m.p: 201-205 °C, ^1^H-NMR (CDCl_3_) δ: 8.28 (1H, s, =NOH), 5.38 (1H, d, *J* = 5.2 Hz, 6-H), 4.56-4.67 (1H, m, 3-H), 2.04 (3H, s, -COCH_3_), 1.89 (3H, s, 21-CH_3_), 1.02 (3H, s, 19-CH_3_), 0.65 (3H, s, 18-CH_3_); ^13^C-NMR (CDCl_3_) δ: 170.6 (3-OCOCH_3_), 158.9 (20-C=NOH), 139.7 (5-C), 122.4 (6-C), 73.9 (3-C), 56.8 (17-C), 56.1, 50.1, 43.8, 38.7, 38.1, 37.0, 36.6, 36.5, 32.0, 31.8, 27.8, 24.2, 23.1, 21.4, 21.0, 19.3, 15.1, 13.1; GC-mass (EI) *m/z*: 342 (M-NOH)^+^, 313 (M-CH_3_COOH)^+^; IR (cm^-1^) : 3315 (20-C=NOH), 1732 (3-OC=OCH_3_), 1651 (20-C=N).

*3β-Acetoxy-5α,6α-epoxy-20-hydroxyiminopregnan-20-one* (**6a**) *and 3β-acetoxy-5β,6β-epoxy-20-hydroxyiminopregnan-20-one* (**6b**): 3*β*-Acetoxy-20-hydroxyimino-5-pregnen-20-one (**5**, 100 mg, 0.27 mmol) and *m*CPBA (139 mg, 0.81 mmol) were dissolved in chloroform (10 mL) and stirred at room temperature for 48 hours. The reaction mixture was treated as the same method of compound **1**. The crude compound was purified by column chromatography (ethyl acetate/*n*-hexane = 1:3) and gave 62 mg (59%) as a mixture of **6a** and **6b**. Compound **6a:** ^1^H-NMR (CDCl_3_) δ: 4.91-4.97 (1H, m, 3-H), 3.09 (1H, d, *J* = 2.0 Hz, 6-H), 2.02 (3H, s, -COCH_3_), 1.86 (3H, s, 21-CH_3_), 1.01 (3H, s, 19-CH_3_), 0.57 (3H, s, 18-CH_3_); ^13^C-NMR (CDCl_3_) δ: 170.6 (3-OCOCH_3_), 158.8 (20-C=NOH), 71.8 (5-C), 63.0 (3-C), 56.7 (6-C), 56.1 (17-C), 50.4, 43.1, 40.2, 39.0, 38.3, 37.6, 36.1, 35.3, 31.8, 29.7, 24.2, 22.6, 23.4, 21.3, 19.5, 15.4. Compound **6b:** ^1^H-NMR (CDCl_3_) δ: 4.87-4.96 (1H, m, 3-H), 2.92 (1H, d, *J* = 4.4 Hz, 6-H), 2.03 (3H, s, -COCH_3_), 1.86 (3H, s, 21-CH_3_), 1.08 (3H, s, 19-CH_3_), 0.60 (3H, s, 18-CH_3_); ^13^C-NMR (CDCl_3_) δ: 170.5 (3-OCOCH_3_), 158.8 (20-C=NOH), 71.9 (5-C), 63.1 (3-C), 56.5 (6-C), 56.1 (17-C), 50.4, 43.1, 40.2, 39.0, 38.3, 37.6, 36.1, 35.3, 31.8, 29.7, 24.2, 22.6, 23.4, 21.3, 19.5, 15.4, 13.1; GC-mass (EI) *m/z*: 358 (M-NOH)^+^, 329 (M-CH_3_COOH)^+^; IR (cm^-1^): 3441 (3-O-H), 1702 (20-C=O).

*3β-Hydroxy-20-hydroxyimino-5-pregnen-20-one* (**7**): To a solution of 5-pregnen-3*β*-ol-20-one (1 g, 3.16 mmol) in ethanol (15 mL) was added hydroxylamine hydrochloride (352 mg, 5.06 mmol) and pyridine (0.5 mL) and the reaction mixture was refluxed at 130 °C for 1 hour. The reaction mixture was cooled down and extracted with dichloromethane and the organic layer was washed with H_2_O, dried with anhydrous MgSO_4_, filtered and concentrated to afford crude oily material. The crude product was purified by column chromatography (ethyl acetate/*n*-hexane = 1:3) affording 1.0 g (95%) as the pure white product **7**, m.p : 220-222 °C, ^1^H-NMR (CDCl_3_) δ : 7.80 (1H, s, = NOH), 5.36 (1H, t, *J* = 2.6 Hz, 6-H), 3.50-3.56 (1H, m, 3-H), 1.89 (3H, s, 21-CH_3_), 1.01 (3H, s, 19-CH_3_), 0.65 (3H, s, 18-CH_3_); ^13^C-NMR (CDCl_3_) δ: 159.0 (20-C=NOH), 140.8 (5-C), 121.5 (6-C), 71.8 (3-C), 56.7 (17-C), 56.2, 50.1, 43.8, 42.3, 38.7, 37.3, 36.5, 32.0, 31.8, 31.6, 24.2, 23.1, 21.0, 19.4, 15.1, 13.1; GC-mass (EI) *m/z*: 331 (M)^+^, 316 (M-CH_3_)^+^, 298 (M^+^-CH_3_-H_2_O); IR (cm^-1^): 3239 (O-H) 1662 (20-C=N).

*20-Hydroxyimino-4-pregnene-3,20-dione* (**8**): The mixture of compound **7** (1 g, 3.01 mmol), aluminum isopropoxide (3 g, 15 mmol) and cyclohexanone (30 mL) was refluxed at 170 °C for 4 hours. The excess of cyclohexanone was distilled off and the residue was added H_2_O and acidified with 5 M HCl and extracted with ethyl acetate. The organic layer was dried with anhydrous MgSO_4_, filtered and evaporated to give crude oil, which was purified by column chromatography (ethyl acetate:*n*-hexane = 1:3) to yield 460 mg (46%) of the pure pale yellow product **8**, m.p: 213-216 °C, ^1^H-NMR (CDCl_3_) δ: 8.14 (1H, s,=NOH), 5.74 (1H, s, 4-H), 1.89 (3H, s, 21-CH_3_), 1.18 (3H, s, 19-CH_3_), 0.68 (3H, s, 18-CH_3_); ^13^C-NMR (CDCl_3_) δ: 199.6 (3-C=O), 171.3 (20-C=NOH), 158.7 (5-C), 123.9 (4-C), 56.6 (17-C), 55.3, 53.8, 43.8, 38.6, 38.5, 35.8, 35.7, 34.0, 32.8, 31.9, 24.1, 23.0, 21.0, 17.4, 15.1, 13.2; GC-mass (EI) *m/z*: 329 (M)^+^, 312 (M-OH)^+^; IR (cm^-1^): 3498 (20-C=NOH), 1658 (3-C=O), 1614 (20-C=N).

*20-Hydroxyimino-1,4-pregnadiene-3,20-dione* (**9**): To a solution of compound **8** (200 mg, 0.6 mmol) in 1,4-dioxane (12 mL) was added DDQ (272 mg, 1.20 mmol), bis(trimethylsilyl)trifluoroacetamide (0.3 mL) and the reaction mixture was refluxed at 150 °C for 12 hours. The reaction mixture was filtered, concentrated and the crude product was purified by column chromatography (ethyl acetate/*n*-hexane = 1:3) to yield 96 mg (49%) of the pure white product **9**. m.p: 105-110 °C, ^1^H-NMR (CDCl_3_) δ: 7.06 (1H, d, *J* = 10.4 Hz, 1-H), 6.24 (1H, dd, *J*_1_ = 1.8 Hz, *J*_2_ = 10.2 Hz, 2-H), 6.08 (1H, s, 4-H), 2.52 (1H, t, *J* = 9.0 Hz, 17-H), 2.12 (3H, s, 21-CH_3_), 1.24 (3H, s, 19-CH_3_), 0.70 (3H, s, 18-CH_3_); ^13^C-NMR (CDCl_3_) δ : 209.2 (20-C=O), 186.3 (3-C=O), 168.9 (5-C), 155.6 (1-C), 127.6 (2-C), 124.0 (4-C), 63.4 (17-C), 55.6, 52.2, 44.1, 43.5, 38.5, 35.5, 33.5, 32.8, 31.5, 24.6, 22.84, 22.82, 18.7, 13.4; GC-Mass (EI) *m/z*: 327 (M)^+^; IR (cm^-1^): 3315 (20-C=NOH), 1662 (3-C=O), 1619 (20-C=N).

*20-Hydroxyimino-5-oxo-A-nor-3,5-secopregnan-3-oic acid* (**10**): 20-Hydroxyimino-4-pregnene-3,20-dione (**8**, 250 mg, 0.76 mmol) was dissolved in *tert*-butanol (5 mL) and anhydrous sodium carbonate (118 mg, 1.11 mmol), H_2_O (2 mL) was added and refluxed at 180 °C. Sodium periodate (1 g, 5.32 mmol) and potassium permanganate (15 mg, 0.09 mmol) dissolved in H_2_O (5 mL) was slowly added to a refluxing reaction mixture, and refluxed for 3 hours. The mixture was filtered and washed with H_2_O and the filtrate was acidified with 5 M HCl and extracted with dichloromethane. The organic layer was dried with anhydrous MgSO_4_, filtered and concentrated to give crude product, which was purified by column chromatography (methanol: dichloromethane = 1:9) to yield 154 mg (58%) of the pure pale yellow product **10**. m.p: 152-155 °C, ^1^H-NMR (CDCl_3_) δ: 2.57 (1H, t, *J* = 8.2 Hz, 17-H), 2.13 (3H, s, 21-CH_3_), 1.13 (3H, s, 19-CH_3_), 0.69 (3H, s, 18-CH_3_); ^13^C-NMR (CDCl_3_) δ: 214.7 (5-C=O), 209.2 (3-COOH), 177.8 (20-C=NOH), 63.4 (17-C), 55.9, 50.4, 47.9, 44.0, 38.4, 38.0, 34.9, 31.5, 31.2, 29.3, 29.0, 24.4, 22.8, 21.5, 20.3, 13.4; GC-Mass (EI) *m/z*: 349 (M)^+^, 332 (M-H_2_O) ^+^; IR (cm^-1^): 3445 (O-H), 1702 (C=O), 1651 (20-C=N).

*4-Aza-20-hydroxyimino-5-pregnene-3,20-dione* (**11**): A mixture of 20-hydroxyimino-4-pregnen-3-one (**10**, 65 mg, 0.18 mmol), ammonium acetate (42 mg, 0.54 mmol) and acetic acid (2 mL) was refluxed for 1 hour. The mixture was evaporated to remove acetic acid and the residue was neutralized with saturated NaHCO_3_ and extracted with dichloromethane. The organic layer was dried with anhydrous MgSO_4_, filtered and concentrated to give crude pale-brown solid, which was recrystallized with ethyl acetate and *n*-hexane and eluted 30 mg (50%) as the pure white product **11**. m.p: 225-227 °C, ^1^H-NMR (CDCl_3_) δ: 7.41 (1H, s, -NH), 4.82 (1H, dd, *J*_1_ = 2.2 Hz, *J*_2_ = 5.0 Hz, 6-H), 2.55 (1H, t, *J* = 8.0 Hz, 17-H), 2.14 (3H, s, 21-CH_3_), 1.10 (3H, s, 19-CH_3_), 0.66 (3H, s, 18-CH_3_); ^13^C-NMR (CDCl_3_) δ: 209.4 (3-C=O), 169.3(20-C=NOH), 140.0 (5-C), 103.0 (6-C), 63.5 (17-C), 56.6, 47.8, 44.0, 38.5, 34.3, 31.8, 31.6, 31.5, 29.6, 28.4, 24.4, 22.8, 21.0, 18.7, 13.3; GC-Mass (EI) *m/z*: 330 (M)^+^, 315 (M-NH)^+^, 300 (M^+^+1-NOH); IR (cm^-1^): 3187 (20-C=NOH), 3071 (N-H), 1669 (3-C=O).

*1,4,6-Pregnatriene-3,20-dione* (**12**): To a mixture of 5-pregnen-3*β*-ol-20-one (3 g, 9.46 mmol) in 1,4-dioxane (40 mL) was added DDQ (8.6 g, 37.90 mmol) at room temperature and the mixture was refluxed for 4 hours. The reaction mixture was cooled down and filtered, washed with 1,4-dioxane and concentrated to give crude product, which was purified by column chromatography (ethyl acetate/*n*-hexane = 1:3) to yield 2 g (70%) of the pure white product **12**. m.p: 145-147 °C (lit. [23] 142-144 °C). 

*1,2-Epoxy-4,6-pregnadiene-3,20-dione* (**13**): A mixture of 1,4,6-pregnatriene-3,20-dione (**12**, 500 mg, 1.61 mmol) in methanol (25 mL) was slowly added 30% hydrogen peroxide (4.5 mL) and 5% NaOH-MeOH (1.2 mL) and stirred at room temperature for 5 hours. The reaction mixture was mixed with H_2_O (5 mL) and concentrated to form the crude precipitate, which was recrystallized with methanol and H_2_O to give 360 mg (68%), of a white product, m.p: 184-187 °C, ^1^H-NMR (CDCl_3_) δ: 6.12 (1H, dd, *J*_1_ = 2.8 Hz, *J*_2_ = 10.0 Hz, 7-H), 6.05 (1H, d, *J* = 10.8 Hz, 6-H), 5.67 (1H, s, 4-H), 3.60 (1H, d, *J* = 4.0 Hz, 2-H), 3.46-3.47 (1H, m, 1-H), 2.60 (1H, t, *J* = 9.4 Hz, 17-H), 2.15 (3H, s, 21-CH_3_), 1.19 (3H, s, 19-CH_3_), 0.74 (3H, s, 18-CH_3_); ^13^C-NMR (CDCl_3_) δ : 209.0 (20-C=O), 194.6 (3-C=O), 158.5 (5-C), 139.6 (7-C), 128.1 (6-C), 119.7 (4-C), 63.1 (17-C), 59.4 (2-C), 54.7 (1-C), 53.5, 45.9, 44.3, 38.8, 38.3, 37.4, 31.6, 23.9, 22.9, 21.2, 18.5, 13.2; GC-mass (EI) *m/z*: 326 (M)^+^; IR (cm^-1^): 1700 (20-C=O), 1662 (3-C=O).

### 3.2. Biology

We purchased LNCaP (androgen receptor dependent cell line) and PC-3 (androgen receptor independent cell line) prostate cancer cells from the Korean Cell Line Bank (Seoul). Finasteride was purchased from Aldrich Co and was used as positive control. RPMI medium 1640, fetal bovine serum (FBS, Gibco), penicillin-streptomycin, phosphate buffered saline (PBS) pH 7.4, 0.25% trypsin EDTA, dimethyl sulfoxide (DMSO, Sigma), thiazolyl blue tetrazolium bromide (MTT, Sigma) and finasteride (Sigma) were purchased.

#### 3.2.1. MTT assay on LNCaP and PC-3 cell

LNCaP and PC-3 prostate cancer cells were grown in RPMI medium 1640 containing charcoal stripped-dextran treated heat inactivated 10% fetal bovine serum (FBS) (Gibco BRL, Rockville, MD, USA) supplemented with 1% penicillin-streptomycin. Cells (2 × 10^3^) were planted in 96 well cell plates and incubated in 5% CO_2_ incubator (NAPCO Water-Jacketed CO_2_ incubator) for 24 hours at 37 °C. Then cells were treated with 0, 1, 5, 10, 20, 50, 100 μM concentrations of sample (5 μL/well) and incubated in 5% CO_2_ incubator for 48 hours at 37 °C. Triplicate wells were used for each concentration. After incubation for 48 hours, 50 μL/well of MTT (2 mg/mL in PBS) was added and further incubated for four hours in the dark. After removal of MTT solution, 150 μL/well DMSO was added to dissolve purple crystal for 15 minutes, after which the absorbance of the solution was measured at 540 nm with an ELISA reader (ELx 808, BIO-TEK). The inhibition effect of LNCaP and PC-3 cell growth was expressed relative cell survival percentage. 

#### 3.2.2. Screening assay for type II 5α-reductase inhibitory activity 

According to the method reported [22], human embryonic kidney cells (HEK293) over-expressing type II 5α-reductase were grown in Dulbecco’s modified Eagle’s medium (DMEM) supplemented with 10% FBS. Stable HEK293 cells (1 × 10^6^) were planted in 24-well cell plate and incubated in 5% CO_2_ incubator for 24 hours at 37 °C. Stable cells were suspended in 50 mM sodium phosphate buffer (pH 5.5) and lysed by sonication for 1 min. The reaction mixture for type II 5-reductase contained 10 μL (10 μg total protein) of cell extract, 65 μL of reaction buffer [60 mM sodium phosphate, pH 5.5, 50 mM KCl, 1 mM NADPH, and 1 μCi [1,2,6,7-^3^H]testosterone (65.0 Ci/mmol, Amersham)], and 1 μM and 10 μM of synthesized compounds. The reaction mixtures were incubated at 37 °C for 1 h, followed by steroids extraction with 250 μL of stop solution (70% cyclohexane, 30% ethyl acetate, 40 μg/mL T and 40 μg/mL DHT). Solvent was dried and steroids were dissolved with 20 μL of chloroform, spotted onto thin layer chromatography (TLC) plate (Merck, Darmastadt, Germany) and developed in 80% toluene, 20% acetone. TLC plate was then exposed to Hyperfilm ^3^H (Amersham, RPN 535B) for three days.

### 3.3. Statistical analysis 

Triplicate analyses were performed for MTT test and all the data were subjected to analysis of variance using ANOVA and Duncan’s multiple range test for significant difference comparison. 

## 4. Conclusions 

Thirteen epoxy- and/or 20-oxime pregnane, pregnene and pregnadiene derivatives were synthesized and evaluated for cytotoxicity activity and 5AR2 inhibition effects. The 5AR2 inhibition screening test results suggested that compounds **5**, **8**, **11** and **12** were potential inhibitors of 5α-reductase type II**.** Especially, **11** was active in the 5AR2 inhibitory test, and inhibited cell proliferation of androgen-dependent cell and **8** was the most active in the 5AR2 inhibitory test, but interestingly, it inhibited PC-3 cells more potently than LNCaP cells. Compound **13** showed the highest cytotoxic effects on both LNCap and PC-3 prostate cancer cells.

## Acknowedgements

Financial support from Catholic University of Daegu is gratefully acknowledged.

## Figures and Tables

**Table 1:** The IC_50_ values of synthesized compounds on LNCaP and PC-3 cells.

Compound No.	LNCaP cell IC_50_ (μM)	PC-3 cell IC_50_ (μM)
Finasteride	>100	>100
**1**	>100	>100
**3**	>100	>100
**7**	>100	>100
**8**	>100	68.95
**11**	71.85	>100
**12**	>100	>100
**13**	15.17	11.83
